# Fornix Rupture in Duplex Kidney due to Internal Iliac Artery Aneurysm

**DOI:** 10.1155/2016/5042456

**Published:** 2016-02-16

**Authors:** Phitsanu Mahawong, Tanop Srisuwan, Kittipan Rerkasem

**Affiliations:** ^1^Division of Urology, Department of Surgery, Faculty of Medicine, Chiang Mai University, Chiang Mai 50200, Thailand; ^2^Division of Diagnostic Radiology, Department of Radiology, Faculty of Medicine, Chiang Mai University, Chiang Mai 50200, Thailand; ^3^Division of Vascular Surgery, Department of Surgery, Faculty of Medicine, Chiang Mai University, Chiang Mai 50200, Thailand

## Abstract

A 70-year-old man presented with severe pain on the right side of the abdomen for 7 days. An abdominal CT angiographic scan showed an impending rupture of a large right internal iliac artery aneurysm which compressed to a right ureter causing hydroureteronephrosis. Fornix rupture of a right duplex kidney was also detected. Selective embolization of right gluteal arteries and then ligation of the right internal iliac artery and right ureterotomy with double J stenting were performed. At the 4-month follow-up appointment, an abdominal ultrasound demonstrated a decrease in the size of the aneurysm and no hydroureteronephrosis after the removal of double J stent.

## 1. Introduction

Fornix rupture (FR) is a unique presentation and this condition may be spontaneous or caused by a sudden increase in intrapelvic pressure which was due to acute ureteral obstruction [1]. The urine in the renal pelvis will extravasate via the ruptured renal fornix to the perirenal space. The most common cause of acute ureteral obstruction is ureteral calculi and most can be effectively managed by endoscopic treatment [2]. We report a very rare cause of FR and the course of treatment.

## 2. Case Presentation

A 70-year-old man with hypertension, coronary artery disease, renal insufficiency, and gouty arthritis presented with a 7-day history of severe intermittent right side abdominal pain. On examination, his blood pressure was 168/114 mmHg and other vital signs were normal. The abdomen was generally distended with marked tenderness of the right lower quadrant. Bowel sound was absent and rebound tenderness was negative. Peripheral arterial pulsations of lower extremities were normal, except both dorsalis pedis pulsations which were absent.

A complete blood count showed a hemoglobin level of 12.4 g/dL, hematocrit of 37.5%, leukocyte of 9,200/*μ*L (neutrophils 95.2%, lymphocytes 3.9%), and platelet count of 265,000/*μ*L. Other laboratory investigations were as follows: nonfasting plasma glucose: 175 mg/dL, blood urea nitrogen: 21 mg/dL, serum creatinine: 1.9 mg/dL, serum albumin: 2.7 gm/dL, serum globulin: 3.2 gm/dL, prothrombin time: 17.5 sec, international ratio: 1.6, and partial thromboplastin time: 31 sec. Serum electrolytes and urinalysis were normal. 1 day later, the hematocrit dropped to 30.8%, but the vital signs and the abdominal pain remained stable.

**Figure 1:**
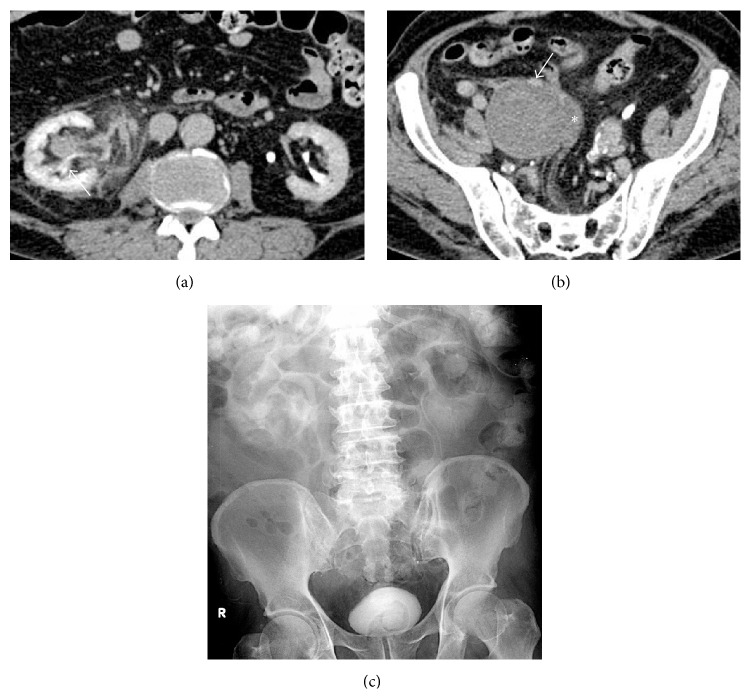
(a) The abdominal CT scan demonstrates fornix rupture with urine extravasation (arrow) around the right kidney and ureter. (b) A saccular right internal iliac artery aneurysm with thick mural thrombus compressing the right midureter (arrow) was noted. (c) Plain abdomen after CT scan shows right perinephric contrast media collection.

**Figure 2:**
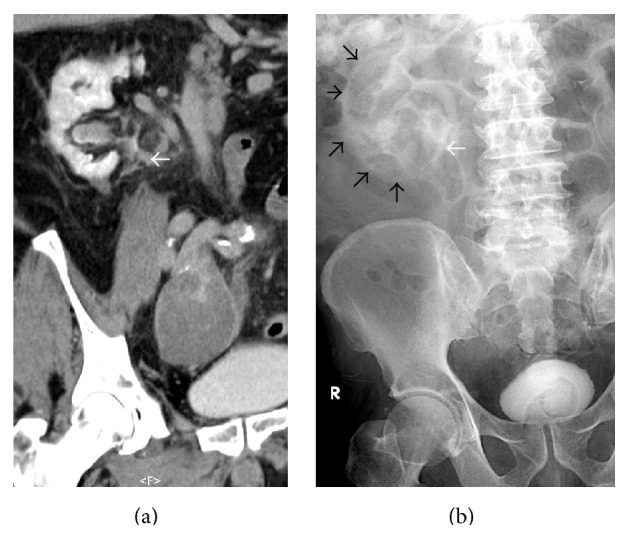
(a) The multiplanar reformation of the CT in excretory phase of the abdomen shows a collection of excreted contrast mixed with urine (arrow) in the soft tissue at renal hilar and parahilar regions. (b) The plain KUB radiograph after CT scan shows the correlated area of contrast leakage (white arrow) seen from CT scan and also additional perinephric contrast collection (black arrows).

**Figure 3:**
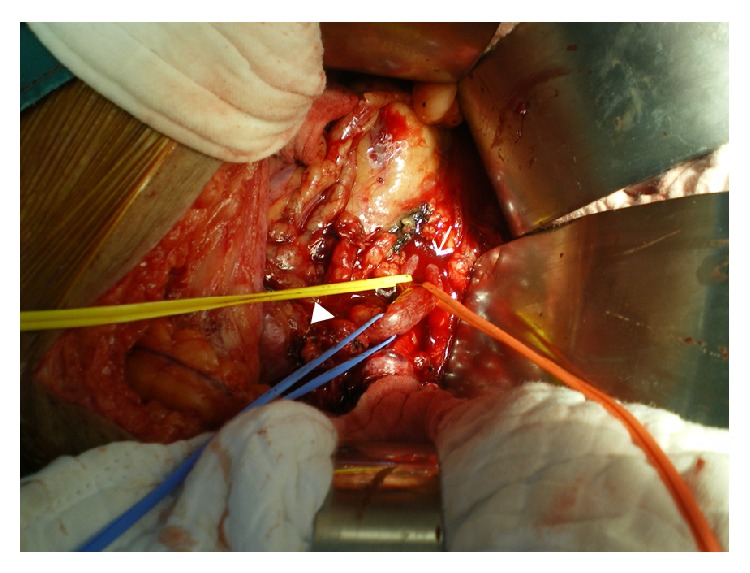
The bifurcation of the right dilated ureter (arrow) was found above the right internal iliac artery aneurysm (arrow head).

An abdominal CT angiographic scan showed an impending rupture of a large right internal iliac artery aneurysm (IIAA) with mass effect to the right midureter causing hydroureteronephrosis and FR (Figure 1). Also a left common iliac and a left internal iliac artery aneurysm were detected. Plain radiograph after CT scan demonstrated right perinephric contrast media collection (Figure 2). The patient underwent selective embolization of anterior and posterior divisions of right internal iliac artery for distal control and then proximal control with right internal iliac artery ligation. Intraoperative findings were the large right IIAA directly compressed to the middle part of the right ureter. The bifid ureter was dilated above the level of compression and there was a significant amount of urine collected in the right retroperitoneal area with marked inflammation (Figure 3).

**Figure 4:**
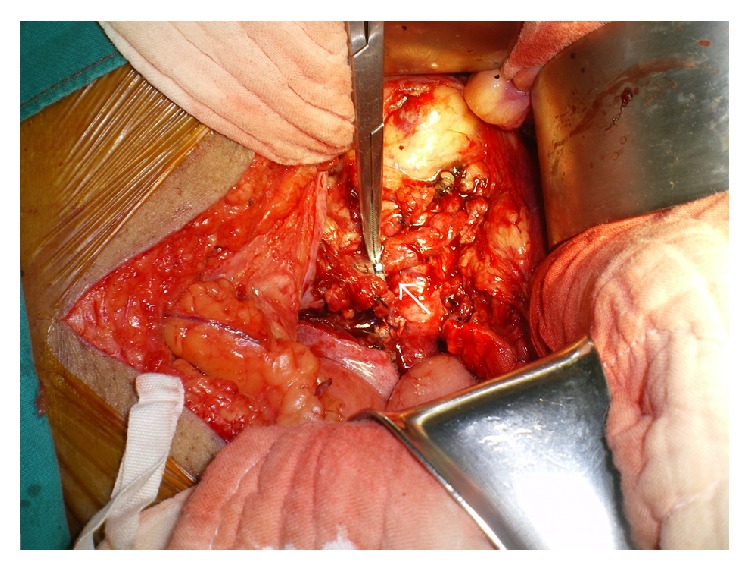
The right ureterotomy was done and the double J stent was inserted (arrow).

After ligation of the right internal iliac artery proximal to the aneurysm, a right ureterotomy was done and a double J stent was placed bypassing the obstructive point of the right ureter (Figure 4). The ureterotomy wound was repaired with chromic catgut 4-0 as interruption fashion. A closed suction drain was placed near the operative field and the abdominal wall was closed layer by layer. A postoperative plain KUB indicated a correct position of the double J stent. The patient remained stable during an early postoperative period and was discharged in the 10th postoperative day without complication.

At 1-month follow-up, an abdominal ultrasound showed normal size and shape of both kidneys and there was no pelvicalyceal dilatation. The stent was endoscopically removed in the 3rd postoperative month. 1 month after the stent removal, intravenous pyelography demonstrated no hydronephrosis and normal excretory function of the right kidney. The patient refused any further treatment of the left iliac artery aneurysms and we plan to tightly control his underlying medical conditions in the future.

## 3. Discussions

Etiologies of FR were reported in literature and the most common cause of the rupture is the acute ureteral obstruction. A ureteral calculus is the major source of the acute obstruction followed by an unknown etiology. Doehn and colleagues reported that up to 60% of 162 FR patients had causes from ureteral calculi [2]. The other infrequent cause of FR is retrograde manipulation of a ureter such as endoscopic ureteral stone extraction [3]. Intravenous pyelography was reported as a cause of FR as well as retrograde cystography [4, 5]. The cause of forniceal rupture may be due to a nongenitourinary origin following an intra-arterial contrast medium application for infrarenal aortic stent placement [6]. Blunt abdominal trauma was also reported as a cause of FR; however the renal parenchyma was intact [7].

To the best of our knowledge, the present case is the first case of FR secondary to ureteral obstruction caused by an IIAA to be published in the English literature. We suppose that the obstruction was an acute process from the impending rupture of the aneurysm. The sudden increase in size of the aneurysm may have caused the acute occlusion to the middle part of the right ureter resulting in FR. Our hypothesis can explain how the pain of the patient was more severe in the lower are than the upper area of the abdomen. Most symptoms and signs of the patient originated from the aneurysm not the FR. In patients in late presentation, we cannot be sure of the cause of the acute abdomen because findings of FR may mimic or mask other causing conditions [8].

In general, most treatments of FR depend on the etiology of FR. Spontaneous cases of FR usually have no symptoms or may be less compliant, so most treatments for this group of FR should be supportive and symptomatic. Analgesics and antibiotics are the major role of conservative management and most of the extravasate urine would be spontaneously absorbed without any invasive procedures. If the perinephric collection is large or the etiologies of ureteral obstructions are significant, they should be corrected appropriately by surgical treatment [9]. The urinary diversion accompanied with the correction of the cause of the obstruction can be done in the same setting or stage [10]. For a traumatic case of FR, it should be corrected by following the guidelines of treatment for kidney injuries. In the present case, we aimed to correct the aneurysm first because of an impending rupture condition and then we performed the internal diversion to ensure the potency of the obstructed ureter. Although this was a duplex kidney obstruction, we demonstrated the favorable outcome of the treatment. We postulate that FR could be from an IIAA and urinary diversion is an acceptable treatment.

## References

[1] Anderhuber F., Reimann R. (1982). Pyelorenal reflux. I. Actual and presumed routes. *Morphologia Medica*.

[2] Doehn C., Fiola L., Peter M., Jocham D. (2010). Causes and course of fornix rupture. *Aktuelle Urologie*.

[3] Bannowsky A. (2008). Iatrogenic fornix rupture caused during retrograde manipulation of the ureter: a case report. *Cases Journal*.

[4] Eggerath A., Friedrichs R. (1985). Evaluation of spontaneous renal extravasation of contrast media in the excretory urogram. *Radiologe*.

[5] Emir L., Karabulut A., Germiyanoğlu C., Erol D. (2001). Calyceal fornix rupture during cystography. *International Urology and Nephrology*.

[6] Niggemann P., Brehmer B., Schuermann K. (2006). Bilateral renal fornix rupture following intraarterial contrast medium application for infrarenal aortic stent placement. *CardioVascular and Interventional Radiology*.

[7] Cass A. S., Lee J. Y., Smith C. S. (1993). Perirenal extravasation with blunt trauma from rupture of a calyceal fornix. *Journal of Trauma*.

[8] Fluckiger R., Gunst M. (1989). Fornix rupture. A contribution to differential acute abdomen diagnosis. *Helvetica Chirurgica Acta*.

[9] Singh I., Joshi M., Mehrotra G. (2009). Spontaneous renal forniceal rupture due to advanced cervical carcinoma with obstructive uropathy. *Archives of Gynecology and Obstetrics*.

[10] Edmonds R. D., Tomaszewski J. J., Jackman S. V., Chaer R. A. (2008). Staged endourologic and endovascular repair of an infrarenal inflammatory abdominal aortic aneurysm presenting with forniceal rupture. *Journal of Vascular Surgery*.

